# Associations between CAG repeat size, brain and spinal cord volume loss, and motor symptoms in spinocerebellar ataxia type 3: a cohort study

**DOI:** 10.1186/s13023-025-03531-8

**Published:** 2025-01-23

**Authors:** Zhi-Xian Ye, Xuan-Yu Chen, Meng-Cheng Li, Xin-Yuan Chen, Yu-Sen Qiu, Ru-Ying Yuan, Zhi-Li Chen, Min-Ting Lin, Jian-Ping Hu, Ying Fu, Wan-Jin Chen, Ning Wang, Shi-Rui Gan, on behalf of the OSCCAR Investigators, Zhi-Xian Ye, Xuan-Yu Chen, Meng-Cheng Li, Xin-Yuan Chen, Yu-Sen Qiu, Ru-Ying Yuan, Zhi-Li Chen, Min-Ting Lin, Jian-Ping Hu, Ying Fu, Wan-Jin Chen, Ning Wang, Shi-Rui Gan

**Affiliations:** 1 Department of Neurology of First Affiliated Hospital, Fujian Medical University, Fuzhou, 350005 China; 2 Institute of Neurology of First Affiliated Hospital, Fujian Medical University, Fuzhou, 350005 China; 3 Institute of Neuroscience, and Fujian Key Laboratory of Molecular Neurology, Fujian Medical University, Fuzhou, 350005 China; 4 Department of Neurology, National Regional Medical Center, Binhai Campus of the First Affiliated Hospital, Fujian Medical University, Fuzhou, 350212 China; 5 School of Basic Medical Sciences, Fujian Medical University, Fuzhou, 350005 China; 6 Department of Radiology of First Affiliated Hospital, Fujian Medical University, Fuzhou, 350005 China; 7 Department of Rehabilitation Medicine of First Affiliated Hospital, Fujian Medical University, Fuzhou, 350005 China

**Keywords:** CAG repeat size, Spinal cord, Sample size, MRI, SCA3

## Abstract

**Background:**

Spinocerebellar ataxia type 3 (SCA3) is a hereditary disease caused by abnormally expanded CAG repeats in the *ATXN3* gene. The study aimed to identify potential biomarkers for assessing therapeutic efficacy by investigating the associations between expanded CAG repeat size, brain and spinal cord volume loss, and motor functions in patients with SCA3.

**Methods:**

In this prospective, cross-observational study, we analyzed 3D T1-weighted MRIs from 92 patients with SCA3 and 42 healthy controls using voxel-based morphometry and region of interest approaches. Associations between expanded CAG repeat size, brain and spinal cord volume loss, and International Cooperative Ataxia Rating Scale (ICARS) scores were investigated using partial correlation and mediation analyses. Sample sizes of potential biomarkers were calculated.

**Results:**

Compared with healthy controls, SCA3 patients had lower cerebellar volume and cervical spinal cord area. SCA3 patients evolved along a stage-independent decline that began in the cerebellum, progressed to spinal cord, brainstem, thalami, and basal ganglia, and extensive subcortex. Expanded CAG repeat size was associated with right cerebellar lobule IV volume (*r* = − 0.423, *P* < 0.001) and cervical spinal cord area (*r* = − 0.405, *P* < 0.001), and higher ICARS (*r* = 0.416, *P* < 0.001). Mediation analysis revealed an indirect effect of expanded CAG repeat size on ICARS through spinal cord. Sample sizes estimation revealed that a minimum sample size was achieved with spinal cord measures.

**Conclusions:**

Our results indicate the potential of cervical spinal cord area as a biomarker for disease progression and a minimum sample size estimation in future clinical studies of SCA3.

**Supplementary Information:**

The online version contains supplementary material available at 10.1186/s13023-025-03531-8.

## Introduction

Spinocerebellar ataxia type 3 (SCA3), also called Machado-Joseph disease, is the most common autosomal dominantly hereditary ataxia disease caused by an expansion of a trinucleotide CAG repeat in the *ATXN3* gene [1]. The CAG repeat size typically falls within the range of 10 to 44 in normal individuals; however, in SCA3 patients, there is a notable expansion, ranging from 56 to 87 [2]. Onset of SCA3 usually occurs in adulthood and is negatively correlated with the expanded CAG repeat size [1]. Clinical symptoms are notably diverse, including gait ataxia, pyramidal and extrapyramidal disorders, and cognitive impairment [3, 4].

Understanding the pathophysiological mechanisms of SCA3 reveals that the presence of abnormal polyglutamine (polyQ)-ataxin-3 aggregates leads to neurotoxic degeneration, affecting neurons and neural networks. This results in structural damage in the cerebellum, brainstem, spinal cord, basal ganglia, and cerebral cortex [1, 5, 6]. Structural damage occurs before the onset of symptoms and is associated with clinical progression in SCA3 [6–9]. Additionally, longitudinal studies have provided evidence supporting the association between expanded CAG repeat size and clinical progression [10, 11].

SCA3 has increasingly become a focal point in intervention studies, with several trials now initiated [12, 13]. The rarity of the disease and the variable rates of disease progression present challenges in assessing the efficacy of therapies. Consequently, a thorough understanding of sensitive and reliable biomarkers that reflect the cascade of pathological events associated with SCA3 is essential.

In this cross-sectional study, we analyzed a cohort of 92 patients with SCA3, all of whom underwent 3D T1-weighted MRI examinations to characterize both clinical and neuroimaging features. Additionally, we aimed to identify the potential biomarker(s) that require the smallest sample size for future targeted therapies by assessing the associations between expanded CAG repeat size, volumetric alterations in the brain and spinal cord, and motor symptoms in SCA3.

## Materials and methods

### Approval of ethical standards and patient consents form

This study received the approval from the Ethics Committee for Medical Research of the Hospital of Fujian Medical University ([2019]195). The ClinicalTrials.gov ID number is NCT04010214. All patients enrolled gave a written informed consent for sharing personal data.

### Study design and enrollment of patients

**Fig. 1:**
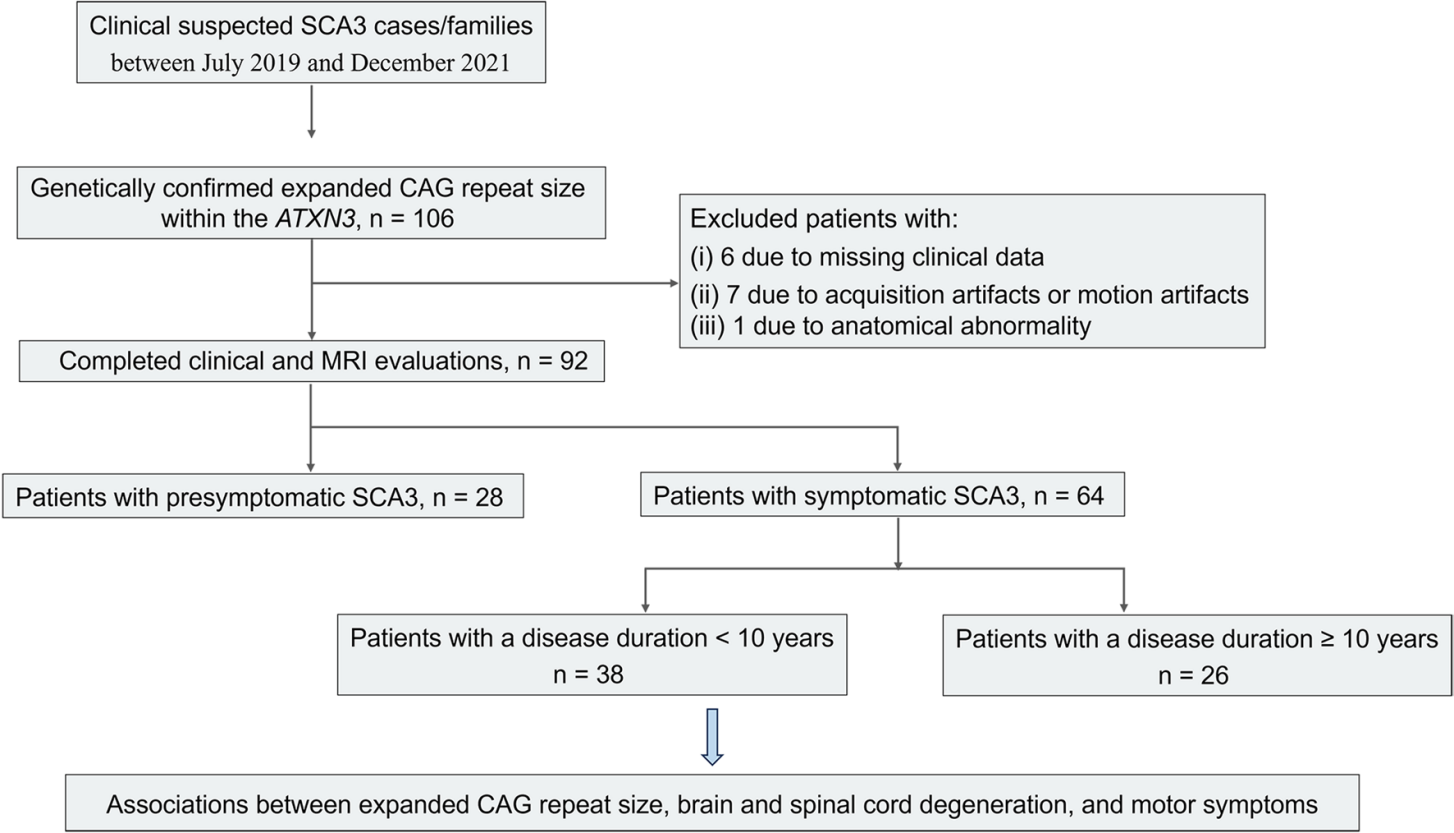
Flowchart of participant in patients with spinocerebellar ataxia type 3 (SCA3)

This prospective, cross-sectional cohort study enrolled participants from the Organization in South-East China for Cerebellar Ataxia Research (OSCCAR). The study participants were patients admitted to the Department of Neurology at the First Affiliated Hospital of Fujian Medical University between July 2019 and December 2021. Eligible 92 patients had genetically confirmed SCA3 with CAG repeats expansion in the alleles of *ATXN3* gene in a range between 56 and 87 [1]. Besides, healthy controls (HCs) composed of 42 sex- and age-matched participants with normal CAG repeat size within the *ATXN3* gene falling in the range from 12 to 44 were included [1]. Each individual had no history of other neurologic or systemic diseases, and other causes of structural lesions on MRIs of brain or spinal cord. The participant flow diagram is illustrated in Fig. 1.

### Clinical assessment

The patients’ demographic characteristics, clinical assessment, and MRI examinations were performed on the same day. For each patient, we collected clinical data regarding age at symptom onset, disease duration, expanded CAG repeat size. All patients’ motor function were assessed using the Scale for the Assessment and Rating of Ataxia (SARA) and the International Cooperative Ataxia Rating Scale (ICARS), which are composite cerebellar ataxia scales [14, 15]. Patients with a SARA score < 3 were defined as presymptomatic SCA3; those with SARA score ≥ 3 were defined as symptomatic SCA3 [16]. In addition, symptomatic SCA3 patients were assigned into 2 subgroups: disease duration < 10 years, and disease duration ≥ 10 years [8].

### MRI data acquisition and analysis

Participants underwent 3.0-Tesla brain MRI examinations (Siemens Skyra scanner) at the time of neuropsychological tests. Sagittal anatomical images were acquired using a 3D T1-weighted magnetization-prepared rapid gradient-echo (MP-RAGE) sequence with the following scan parameters: repetition time = 2300 ms, echo time = 2.3 ms, inversion time = 900 ms, flip angle = 8°, field of view = 240 mm × 256 mm, number of slices = 192, voxel size = 1 × 1 × 1 mm^3^, total acquisition time = 5.2 min. Completing the MRI scan, all data sets were checked by one experienced radiologist for quality control, including motion correction and cutoffs for head movement.

Structural MRI data analysis was undertaken using Statistical Parametric Mapping software (SPM12, http://www.fl.ion.ucl.ac.uk/spm) running on MATLAB. The T1-weighted images were segmented into gray matter (GM), white matter (WM), and cerebrospinal fluid, and were normalized to standard space with Difeo-morphic Anatomical Registration using the Exponentiated Lie algebra. Modulated GM and WM images were smoothed using an 8-mm full-width at half-maximum Gaussian filter for further voxel-wise statistical analyses. The total intracranial volume (TIV) was calculated for each subject by adding the volume of GM, WM, and cerebrospinal fluid.

The cerebellum and upper cervical cord (C2 level) were manually segmented by two trained neurologists independently in the sagittal, axial, and coronal planes of the 3D T1-weighted MP-RAGE scans and their volumes were calculated. Inter-rater reliability was assessed, and the final corrected manual segmentation volume was determined by consensus of the two raters. The cerebellar volume was expressed as the ratio of the cerebellar volume to the TIV. The upper cervical cord volume divided their reconstructed slice thickness (3.0 mm) to obtain the upper cervical cord area. Each participant’s mean upper cervical cord area (MUCCA) was calculated by averaging the areas from 5 contiguous axial upper cervical cord slices [17]. A reliable sub-segmentation of the cerebellum into the cerebellar lobules was underwent using CERES software [18].

### Statistics

The normality of the standardized residuals was evaluated with the Shapiro–Wilk’s test. The continuous variables were presented as median (range) and categorical data as number (%). Reliability of cerebellum and upper cervical cord segmentations were assessed with use of the intraclass correlation coefficient (two-way mixed model, single measure, absolute agreement). A univariate analysis for comparing the demographic, clinical, and brain MRI parameters of SCA3 patients and HCs was conducted using the Mann–Whitney *U* or Chi-square test, and multiple linear regression models were employed for the comprehensive analyses of cerebellar volume and MUCCA.

A voxel-based morphometry (VBM) analysis was used to identify differences in the GM and WM volumes between SCA3 patients and HCs using SPM12. Covariates of no interest, including sex, age, and TIV of each participant, were included in the analysis. Statistical significance was established when P values were < 0.05, following family-wise error (FWE) cluster-level correction. To characterize the spatiotemporal patterns of brain and spinal cord volume loss, we compared the structural differences of presymptomatic patients, patients with a disease duration < 10 years, and Patients with a disease duration ≥ 10 years. The associations between CAG repeat size, brain volume(s) and MUCCA, and motor symptoms (analyzed by total ICARS score) were investigated using partial correlation analysis, controlling for potential effects of age, sex, and TIV. Mediation analyses were performed to evaluate whether alterations in brain volume(s) and MUCCA mediate the association between expanded CAG repeat size and ICARS.

The sample size calculation for a hypothetical clinical trial, aimed at evaluating a treatment that could potentially slow disease progression over a 7-year period, was conducted using PASS 15. To ensure comparability, patients were divided into two groups with similar numbers based on disease duration: those with a duration ≥ 7 years and those with a duration < 7 years. Standardized effect sizes were estimated for each clinical variable and quantitative MRI metric independently. The standardized effect sizes |t| were calculated as the differences between the means of two groups, divided by the standard deviation of the group with a longer disease duration. An 80% power level was used and a two-side 0.05 level was considered significant.

Statistical analyses were performed using SPSS v25.0 (IBM, Armond, New York) and Prism v7.0 (GraphPad, San Diego, CA). The *P* < 0.05 were considered statistically significant. Bonferroni correction was applied to adjust results in multiple comparisons.

## Results

### Demographic and clinical characteristics

A total of 92 SCA3 patients were selected and further analyzed in this study. SCA3 patients and HCs were matched for age and sex (*P* > 0.05). Among the SCA3 patients, 64 (69.6%) were symptomatic and 28 (30.4%) were presymptomatic. The median CAG repeat size was 75 (range, 60–81). The main demographic and clinical characteristics are reported in Table 1.

**Table 1:** Demographic and clinical characteristics of SCA3 patients and healthy controls

Parameters	Healthy controls	Whole SCA3	*P*-value
Total *n*	42	92	
Female sex	26 (61.9)	42 (45.7)	0.081
Age at examination, year	35 (14, 65)	39 (15, 58)	0.474
Age at onset, year*	NA	33 (16, 50)	NA
Disease duration, year*	NA	9 (2, 20)	NA
Expanded CAG repeats	NA	75 (60, 81)	NA
SARA scores, points	NA	7.5 (0, 28)	NA
ICARS scores, points	NA	18 (0, 70)	NA
Total intracranial volume, mL	1397 (1232, 1712)	1426 (1145, 1752)	0.434
Cerebellar volume (ratio of TIV **✖** 10^2^)	9.07 (7.79, 10.92)	8.25 (6.15, 10.09)	< 0.001
MUCCA, mm^2^	77.2 (50.0, 98.3)	64.5 (41.0, 84.7)	< 0.001

Numbers are presented as median (range)

Mann–Whitney U test or Pearson χ2 was used to compare variables

*CAG* cytosine-adenine-guanine, *ICARS* International Cooperative Ataxia Rating Scale, *MUCCA* mean upper cervical cord area, *NA* not applicable, *SARA* Scale for the Assessment and Rating of Ataxia, *SCA3* spinocerebellar ataxias type 3, *TIV* total intracranial volume

^*^The two variables were described among the symptomatic patients; n = 64

### Brain volume(s) and MUCCA are lower in patients with SCA3 than in healthy controls

**Fig. 2:**
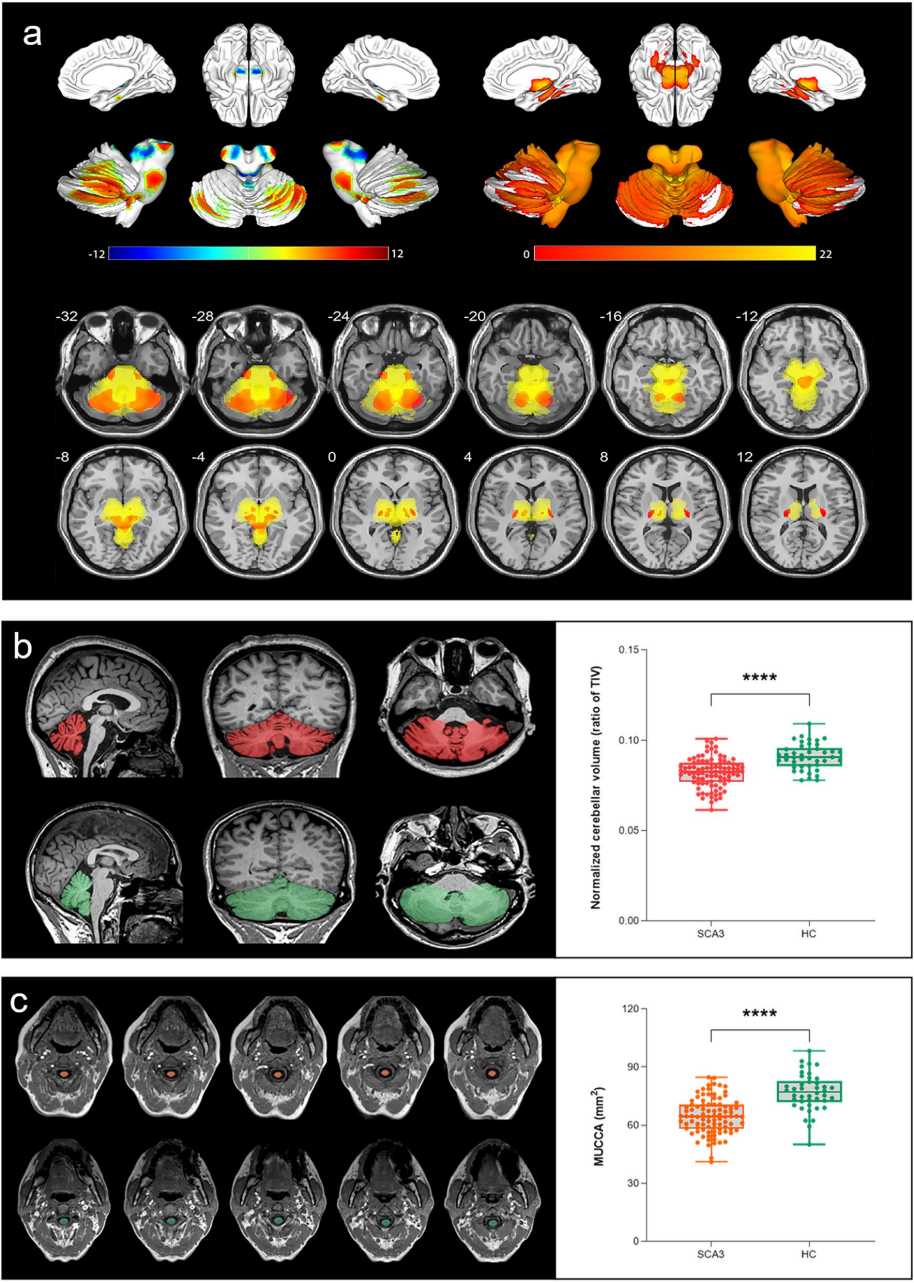
Brain volume(s) and spinal cord area in patients with spinocerebellar ataxia type 3 (SCA3). **a** Voxel-based morphometry analysis showed brain volume differences in grey matter (GM, upper left), white matter (WM, upper right) and GM and WM overlaps (bottom) between SCA3 patients and healthy controls (HC). The results were displayed using the xjView toolbox. Color bar represents t-values. Statistical tests were evaluated at a significance level of *P* < 0.05, the family-wise error corrected at the cluster level. **b** Region of interest analysis showed cerebellar volume differences between SCA3 patients and HC. T1-weighted magnetization-prepared rapid gradient-echo MRI displayed segmentation of cerebellum in sagittal, coronal and axial planes in patient with SCA3 (upper, red) and HC (lower, green). Cerebellar volume was normalized according to total intracranial volume (TIV). Box represents interquartile range and median, whereas whiskers represent minimum and maximum values in data. **** = *P* < 0.0001. **c** Region of interest analysis showed mean upper cervical cord area (MUCCA) differences between SCA3 patients and HC. T1-weighted magnetization-prepared rapid gradient-echo MRI displayed 5 contiguous upper cervical cross sections in axial plane in patient with SCA3 (upper, orange) and HC (lower, green). Box represents interquartile range and median, whereas whiskers represent minimum and maximum values in data. **** = *P* < 0.0001

When assessing volumetric differences between SCA3 patients and HCs, the VBM analysis indicated that SCA3 patients had lower volume in various brain regions including the cerebellum, brainstem, thalami, and bilateral globus pallidum (peak* t* value = 20.8, *P* < 0.05, FWE-corrected). Notably, volume loss was found in both GM and WM regions, with the latter showcasing more extensive impairment (Fig. 2a).

Segmentation of cerebellar volume and MUCCA demonstrated an excellent intraclass correlation coefficient across different operators (0.985; 95%CI [0.975, 0.991]; *P* < 0.001; 0.965; 95%CI [0.884, 0.984]; *P* < 0.001). In univariate analysis, patients with SCA3 showed a lower TIV-normalized cerebellar volume (*P* < 0.001) and lower MUCCA (*P* < 0.001) compared with HCs (Fig, 2b–c). No differences between the SCA3 group and HCs were found in TIV (*P* = 0.434). After adjusting for the sex, age, and TIV, patients with SCA3 still demonstrated lower cerebellar volume (β = − 0.416; regression coefficient = − 13.81, 95%CI [− 17.62, − 9.993]; *P* < 0.001) and MUCCA (β = − 0.497; regression coefficient = − 11.56, 95%CI [− 14.83, − 8.283]; *P* < 0.001), compared with HCs (Table S1).

### Brain volume(s) and MUCCA measures according to disease stage group in patients with SCA3

**Fig. 3:**
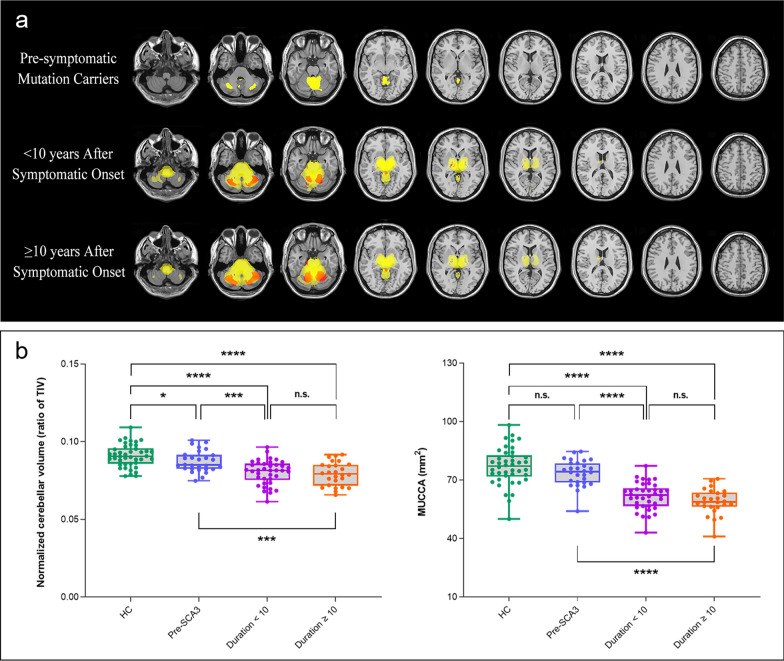
Brain volume(s) and spinal cord area(s) in patients with different stages of spinocerebellar ataxias type 3 (SCA3). **a** Voxel-based morphometry analysis showed grey matter (red) and white matter (yellow) volume differences in patients with presymptomatic SCA3, disease duration < 10 years, and disease duration ≥ 10 years, compared with healthy controls (HC), respectively. The results were displayed using the xjView toolbox. Statistical tests were evaluated at a significance level of *P* < 0.05, the family-wise error corrected at the cluster level. **b** Box plots showed cerebellar volume and mean upper cervical cord area (MUCCA) differences between patients with presymptomatic SCA3 (Pre-SCA3), disease duration < 10 years, and disease duration ≥ 10 years. Cerebellar volume was normalized according to total intracranial volume (TIV). Box represents interquartile range and median, whereas whiskers represent minimum and maximum values in data. * = *P* < 0.05, *** = *P* < 0.001, **** = *P* < 0.0001, n.s. = not significant

The VBM analysis indicated that brain volumes differed among the groups (Fig. 3a). Compared with HCs, presymptomatic SCA3 patients had lower volumes primarily in the cerebellum. Patients with a disease duration < 10 years had lower volume not only in the cerebellum but in the brainstem, thalami, and basal ganglia. Additionally, those with a disease duration ≥ 10 years showed more extensive damage in these regions (all *P* < 0.05, FWE-corrected at the cluster level).

In the region of interest (ROI) analysis, both cerebellar volume and MUCCA differed among the groups (all *P* < 0.001) (Fig. 3b; Table S2; Table S3). Patients with a disease duration ≥ 10 years had lower cerebellar volume (ratio of TIV) and lower MUCCA compared with both the HCs group and presymptomatic patients (*P* < 0.001). Similarly, patients with a disease duration < 10 years had lower cerebellar volume (ratio of TIV) and lower MUCCA compared with both the HCs group and presymptomatic patients (*P* < 0.001). A difference in cerebellar volume was found between the HCs and presymptomatic groups (*P* = 0.032), while no difference in MUCCA was found (*P* = 0.115). Additionally, there was no difference in cerebellar volume or MUCCA between patients with a disease duration < 10 years and those with a duration ≥ 10 years (*P* > 0.05).

### Association of expanded CAG repeat size, brain and spinal cord degeneration, and motor symptoms in patients with SCA3

We further examined potential associations among expanded CAG repeat size, brain volumes, MUCCA, and motor symptoms in SCA3 patients using multivariable-adjusted models, accounting for age, sex, and TIV as covariates.

The VBM analysis indicated that a larger CAG repeat size was associated with a lower volume in the cerebellum, specifically in the right lobule IV_V and right lobule VI (peak* t* value = 4.24, *P* < 0.05, FWE-corrected at the cluster level; Fig. S1). We then individually extracted the volumes of the right lobule IV, right lobule V, and right lobule VI of the cerebellum for each SCA3 patient.

**Fig. 4:**
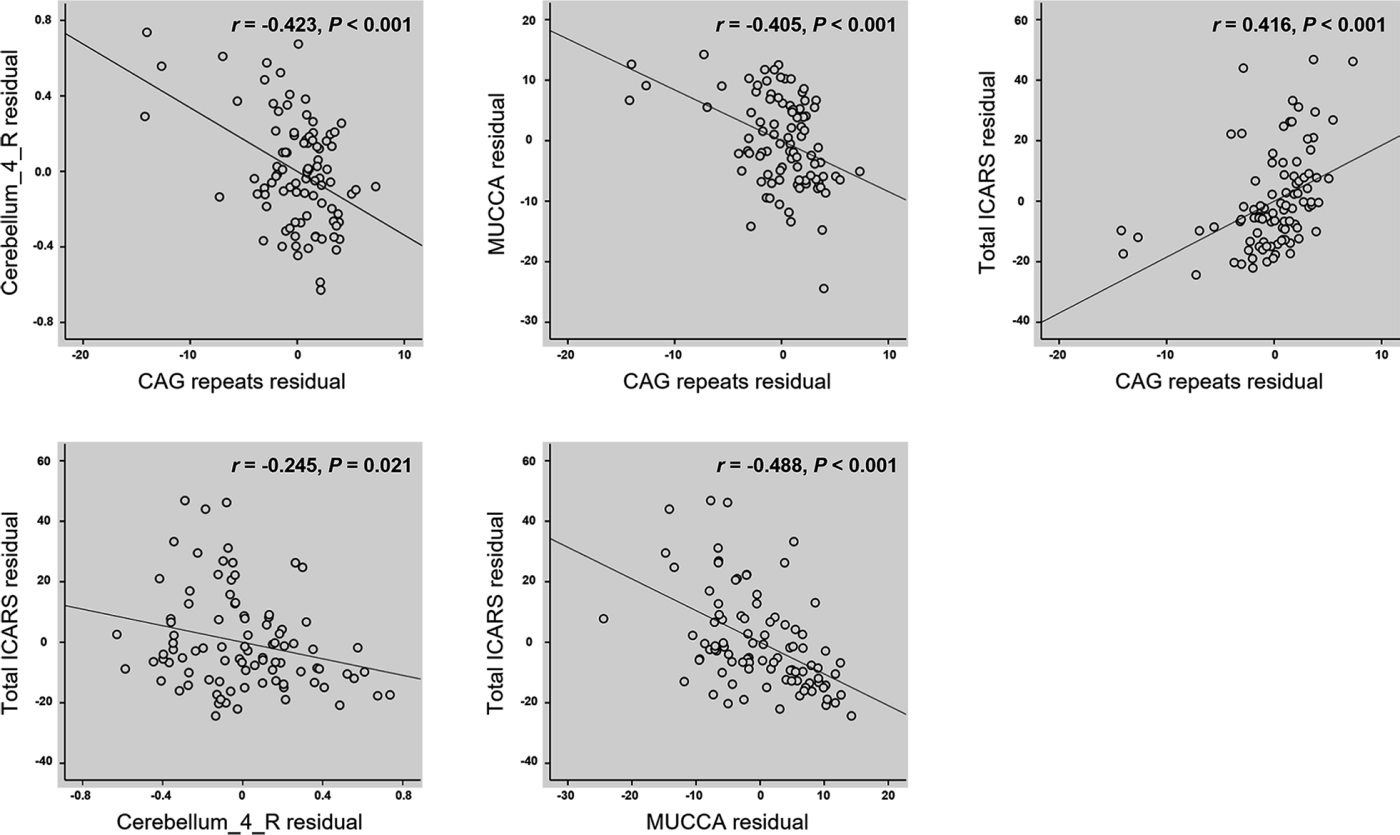
Partial correlation analyses in patients with spinocerebellar ataxias type 3 (SCA3). The CAG repeat size was negatively associated with right cerebellar lobule IV volume and mean upper cervical cord area (MUCCA), and was positively associated with International Cooperative Ataxia Rating Scale (ICARS) score. Both right cerebellar lobule IV volume and MUCCA were negatively associated with ICARS score. Results were presented with the adjusted *r* value, controlling for the effects of age, sex, and total intracranial volume

In the ROI-based partial correlation analysis, we found that a larger CAG repeat size was associated with a lower right lobule IV volume (*r* = − 0.423, *P* < 0.001) and a lower MUCCA (*r* = − 0.405, *P* < 0.001), as well as a higher ICARS score (*r* = 0.416, *P* < 0.001). Additionally, negative correlations were observed between right lobule IV volume (*r* = − 0.245, *P* = 0.021) and MUCCA (*r* = − 0.488, *P* < 0.001) with the ICARS score (Fig. 4). No associations were detected between CAG repeat size and the volumes of either the right lobule V or right lobule VI.

**Fig. 5:**
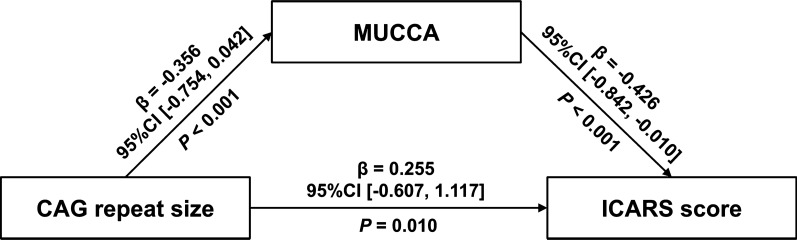
Mediation analysis. The mean upper cervical cord area (MUCCA) was as a mediator of the association between CAG repeat size and International Cooperative Ataxia Rating Scale (ICARS) score while controlling for the effects of age, sex, and total intracranial volume. β, standardized coefficients; CI, confidence interval

Given the observed associations between right cerebellar lobule IV volume, MUCCA, and ICARS, we examined whether alterations in cerebellar right lobule IV volume or MUCCA could serve as potential mediators in the association between expanded CAG repeat size and ICARS, while controlling for age, sex, and TIV as covariates. The expanded CAG repeat size showed a significant total effect on ICARS (β = 0.407; 95%CI [− 0.442, 1.256]; *P* < 0.001). Notably, our findings indicated that MUCCA measures in SCA3 partly mediated the association between expanded CAG repeat size and ICARS (Fig. 5). However, this mediation effect was not observed for the volume of the right lobule IV.

### Sample size estimation

We conducted sample size calculations for disease progression based on the total ICARS score, right cerebellar lobule IV volume, and MUCCA. The comparisons and the standardized effect sizes between patients with a disease duration ≥ 7 years versus those with a duration < 7 years were shown in Table S4. The minimum sample size was estimated with MUCCA as the outcome measure (n = 18), compared with the total ICARS score (n = 32) and the right cerebellar lobule IV volume (n = 160).

## Discussion

In this cross-sectional cohort study, we provided a comprehensive description of the clinical and structural characteristics of SCA3. Our results demonstrated that the spatiotemporal decline in the SCA3 brain began in the cerebellum and progressed to the spinal cord, brainstem, and extensive subcortex. Correlation analysis revealed that the expanded CAG repeat size was associated with volume loss in regional cerebellum and mean upper cervical cord area (MUCCA). Notably, our mediation analysis showed that the association between the expanded CAG repeat size and ICARS was mediated by MUCCA measures.

Previous postmortem and MRI studies have reported severe and widespread brain pathology in SCA3, characterized by neuronal loss in the cerebellum (including both cerebellar lobules and WM), spinal cord, brainstem, thalamus, basal ganglia, and cerebral cortex [5, 6, 19–22]. A neuropathological study of 12 genetically confirmed autopsy cases of SCA3 revealed a pattern of neuronal loss that was less prominence in the cerebellar cortex but more pronounced in the dentate nucleus [22]. Additionally, an MRI study involving 38 SCA3 patients reported volume loss primarily in the cerebellar WM and brainstem compared to GM structures [23]. Consistent with these previous autopsy and smaller-sample studies, we observed significant volume loss predominantly affecting cerebellar WM (including dentate nucleus), brainstem (including medulla, pons, and midbrain), and thalami in patients with SCA3. The differential vulnerability of WM versus GM structures suggests that therapeutic strategies aimed at preserving or restoring WM integrity may help mitigate SCA3 progression.

Previous MRI studies have reported that structural damage in SCA3 preceded the onset of clinical manifestations [7, 8], a pattern also observed in Huntington’s disease and familial amyotrophic lateral sclerosis [24, 25]. In presymptomatic SCA3 patients, volume loss has been identified in the spinal cord, cerebellar WM, and pons [7, 8, 26, 27]. Our findings align with these observations, indicating cerebellar WM volume loss in the presymptomatic stage. Moreover, these studies and ours implicated that structural damage of SCA3 followed a specific temporal process. As disease duration increased, volume loss extended from the cerebellum—affecting cerebellar lobule VI, lobule Crus I, and cerebellar WM—to the brainstem, including medulla, pons, and midbrain, and to the upper cervical spinal cord, and afterwards affecting specific subcortical regions, particularly the thalami and globus pallidum. Beyond SCA3, both Alzheimer’s disease and Parkinson’s disease follow a specific temporal process (Braak’s staging) [28–31]. A recent study examining Alzheimer’s disease reported that microglial activation plays a key role in tau pathology spread across Braak’s stages [31]. Postmortem studies of Parkinson’s disease brains have also identified a close association between microglial activation and Braak’s staging [32]. Previous studies on patients with SCA3 have reported an increase in activated microglia in the brain [33, 34]. Collectively, these studies suggest that the possibility that interactions between expanded ataxin-3 and activated microglia lead to the trans-synaptic spread of expanded ataxin-3, triggering a cascade of events that culminates in the death of nearby neurons.

Previous studies in SCA3 patients regarding the associations between expanded CAG repeat size and brain structure changes were still lack. A potential association between the CAG repeat size and hypothalamic volume was observed in one small-sample SCA3 report [35]. Another study reported the CAG repeat size had no significant influences on the degeneration rate of cerebellar function and structure [36]. Previous pathological data have showed that expanded CAG repeats size was inversely associated with the number of lower motor neurons in the spinal cord [37]. In the present study, we recruited a much larger cohort and our MRI data revealed that a larger CAG repeat size could accelerate volume loss in the right cerebellar lobule IV and cervical spinal cord (as analyzed by MUCCA). In addition, several studies have indicated that expanded CAG repeat size was a distinct determinant of SCA3 progression (as analyzed by ICARS) [11, 38, 39]. We also found the association between expanded CAG repeat size and ICARS in SCA3. Considering that structural damage preceded clinical symptoms, and several studies reported the associations between cervical cord atrophy and clinical parameters, including disease duration and severity [6, 7]. Through mediation analysis, we found that expanded CAG repeat size not only directly influenced the progression of ICARS but also affected it through MUCCA measures. These findings suggested that mutant ataxin-3 toxicity caused by CAG expansion drove cerebellar lobule IV and spinal cord structure damage and subsequent clinical progression.

As targeted therapies for SCA3 are in development (NCT05160558, NCT05822908). Future, preventive trials are a realistic option and a comprehensive understanding of biomarkers that reflect the cascade of pathological events associated with SCA3 is a crucial prerequisite. Our findings indicated that both right cerebellar lobule IV volume and MUCCA were associated with disease duration or ICARS progression in SCA3 patients, suggesting that quantitative analyses of them could serve as valuable biomarkers for disease progression in SCA3. Furthermore, by setting the median disease duration of 7 years as a cut-off value to monitor disease progression under the assumption of a given treatment, we assessed potential biomarkers including total ICARS, right cerebellar lobule IV volume, and MUCCA for sample size estimation. The results suggested that MUCCA required the minimum participants, followed by total ICARS, and the right cerebellar lobule IV volume requiring highest sample size. Therefore, we propose MUCCA as potential candidate for clinical trial endpoints. Such estimations are crucial for therapeutic trials against SCA3 disease, where patient recruit and follow-up are challenging, making large sample sizes impractical.

Several limitations should be noted in our study. First, our findings were based on cross-sectional data, which do not allow an accurate estimation of the time-dependent evolution of brain and spinal cord degeneration. Second, our study focused solely on measuring cervical spinal cord cross-sectional area. Future investigations could enhance the understanding of the association between expanded CAG repeat size, spinal cord degeneration, and ICARS by examining the entire length of the spinal cord. Third, the reliability of these biomarkers in reflecting disease progression needs confirmation through longitudinal studies. Also, since our study was conducted in a single center, the results should be replicated in other studies for broader validation. Finally, given that our study was based on neuroimaging of structural MRI, it would be ideal if future investigations can seek confirmation based on postmortem histopathology.

## Conclusions

In this cohort study, our results revealed that expanded CAG repeat size was associated with volume loss in the cerebellum and cervical cord, as well as with motor function in patients with SCA3. Further longitudinal research is warranted to unravel the interactions between heredity, clinical factors, and neurodegeneration. Additionally, measuring the cervical cord cross-sectional area might be a potentially sensitive biomarker with moderate duration to track disease changes in future studies.

## Supplementary Information

Additional file 1.

## Data Availability

A fully anonymized version of the dataset used for analysis with individual participant data and a data dictionary will be available for other researchers, via https://research.yiducloud.com.cn/#/project/list?desease=research_nervous_standard. Written proposals will be assessed by corresponding author of this study (ganshirui@fjmu.edu.cn) and a decision made about the appropriateness of the use of data.

## Abbreviations

FWE: Family-wise error
GM: Gray matter
HCs: Healthy controls
ICARS: International cooperative ataxia rating scale
MP-RAGE: Magnetization-prepared rapid gradient-echo
MUCCA: Mean upper cervical cord area
ROI: Region of interest
SARA: Scale for the assessment and rating of ataxia
SCA3: Spinocerebellar ataxia type 3
TIV: Total intracranial volume
VBM: Voxel-based morphometry
WM: White matter
