# Supplementary material for: Associations between CAG repeat size, brain and spinal cord volume loss, and motor symptoms in spinocerebellar ataxia type 3: a cohort study
Source: Orphanet J Rare Dis. 2025 Jan 23;20:35. doi: 10.1186/s13023-025-03531-8 (PMC11761751; doi:10.1186/s13023-025-03531-8)

**Journal name: Orphanet Journal of Rare Diseases**

**Associations between CAG repeat size, brain and spinal cord volume loss, and motor symptoms in spinocerebellar ataxia type 3: a cohort study**

Zhi-Xian Ye, MSc, Xuan-Yu Chen, MSc, Meng-Cheng Li, MSc, Xin-Yuan Chen, MD, Yu-Sen Qiu, MD, Ru-Ying Yuan, MSc, Zhi-Li Chen, MSc, Min-Ting Lin, MSc, Jian-Ping Hu, MD, PhD, Ying Fu, MD, PhD, Wan-Jin Chen, MD, PhD, Ning Wang, MD, PhD, Shi-Rui Gan, MD, PhD

**Corresponding to:**

Shi-Rui Gan, Department of Neurology of First Affiliated Hospital, Fujian Medical University, Fuzhou 350005, China. E-mail: ganshirui@fjmu.edu.cn; Tel: 086-591-87982772.

**Contents**

**1 Appendix**

1.1 Additional files

1.1.1 Table S1 Multiple regression analyses of cerebellar volume and spinal cord area between different groups

1.1.2 Table S2 Volumetric MRI measures according to disease duration group in all SCA3 patients

1.1.3 Table S3 Comparison results (*P* values) of MRI measures according to SCA3 disease stage group

1.1.4 Table S4 Comparisons and standardized effect size between SCA3 patients with disease duration ≥ 7 years and < 7 years

1.1.5 Figure S1 Voxel-based correlation analysis between CAG repeat size and brain volume(s) in patients with spinocerebellar ataxias type 3 (SCA3).

**1.1 Additional files**

| Table S1 Multiple regression analyses of cerebellar volume and spinal cord area between different groups | | | | |  |
| --- | --- | --- | --- | --- | --- |
|  | **Beta** | **Coefficient, B** | **95% CI for B** | ***P*-value** | |
| **Cerebellar volume: SCA3 vs. HCs** | | | | | |
| Sex | 0.096 | 2.950 | -1.913, 7.814 | 0.232 | |
| Age | -0.316 | -0.420 | -0.571, -0.270 | <0.001 | |
| TIV | 0.415 | 0.047 | 0.030, 0.064 | <0.001 | |
| Between-group difference | -0.416 | -13.81 | -17.62, -9.993 | <0.001 | |
| **MUCCA: SCA3 vs. HCs** | | | | | |
| Sex | -0.013 | -0.281 | -4.455, 3.893 | 0.894 | |
| Age | -0.265 | -0.248 | -0.377, -0.118 | <0.001 | |
| TIV | 0.223 | 0.018 | 0.003, 0.033 | 0.021 | |
| Between-group difference | -0.497 | -11.56 | -14.83, -8.283 | <0.001 | |
| Variables comparisons between different groups were done using multiple regression models.  Abbreviations: HCs = healthy control individuals; MUCCA = mean upper cervical cord area; SCA3 = spinocerebellar ataxias type 3; TIV = total intracranial volume. | | | | | |

| Table S2 Volumetric MRI measures according to disease duration group in all SCA3 patients | | | |
| --- | --- | --- | --- |
| **Characteristics** | **Presymptomatic SCA3** | **Duration < 10 years** | **Duration ≥ 10 years** |
| Number | 28 | 38 | 26 |
| Female sex | 13 (46.4) | 19 (50.0) | 10 (38.5) |
| Age at examination, year | 29 (15–47) | 41 (24–58) | 45 (27–57) |
| Age at onset, year | NA | 34 (16–50) | 31 (17–43) |
| Disease duration, year | NA | 7 (2–9) | 13 (10–20) |
| Expanded CAG repeats | 74 (60–80) | 75 (70–80) | 75.5 (66–81) |
| SARA scores, points | 0 (0–2) | 8.25 (4–23) | 10.5 (3–28) |
| ICARS scores, points | 1 (0–12) | 21 (6–58) | 29 (4–70) |
| Total intracranial volume, mL | 1412 (1145–1664) | 1461 (1151–1739) | 1416 (1194–1752) |
| Cerebellar volume  (ratio of TIV ✖ 10^2^) | 8.50 (7.48–10.09) | 8.16 (6.15–9.65) | 7.93 (6.57–9.18) |
| MUCCA, mm2 | 74.3 (54.0–84.7) | 62.3 (43.0–77.3) | 58.8 (41.0–77.3) |
| Note: numbers are presented as median (range). Abbreviations: CAG = cytosine-adenine-guanine; ICARS = International Cooperative Ataxia Rating Scale; MUCCA = mean upper cervical cord area; NA = not applicable; SARA = Scale for the Assessment and Rating of Ataxia; SCA3 = spinocerebellar ataxias type 3; TIV = total intracranial volume. | | | |

| Table S3 Comparison results (*P* values) of MRI measures according to SCA3 disease stage group | | | | |
| --- | --- | --- | --- | --- |
| ***P* value** | **All groups** | **Presymptomatic  vs. Duration < 10 years** | **Presymptomatic  vs. Duration ≥ 10 years** | **Duration < 10 years vs. Duration ≥ 10 years** |
| Total intracranial volume, mL | 0.846 | 0.638 | 0.966 | 0.510 |
| Cerebellar volume  (ratio of TIV * 10^2^) | <0.001 | <0.001 | <0.001 | 0.528 |
| MUCCA, mm2 | <0.001 | <0.001 | <0.001 | 0.095 |
| Note: all values are *P* values. Bonferroni correction for multiple comparison. Abbreviations: MUCCA = mean upper cervical cord area; SCA3 = spinocerebellar ataxias type 3; TIV = total intracranial volume. | | | | |

| Table S4 Comparisons and standardized effect size between SCA3 patients with disease duration ≥ 7 years and < 7 years | | | |  |
| --- | --- | --- | --- | --- |
| **Variables** | **SCA3 patients with disease duration < 7 years (n = 46)** | **SCA3 patients with disease duration ≥ 7 years (n = 46)** | **Standardized effect size \|t\|** | |
| Total ICARS | 10.70 ± 12.094 | 28.92 ± 17.285 | 1.055 | |
| Right cerebellar lobule IV volume | 2.14 ± 0.391 | 1.98 ± 0.348 | 0.447 | |
| MUCCA | 69.25 ± 9.240 | 60.06 ± 6.102 | 1.506 | |
| Note: numbers are presented as mean (SD). Abbreviations: ICARS = International Cooperative Ataxia Rating Scale; MUCCA = mean upper cervical cord area; SCA3 = spinocerebellar ataxias type 3 | | | |  |

**Figure S1 Voxel-based correlation analysis between expanded CAG repeat size and brain volume(s) in spinocerebellar ataxias type 3 (SCA3).** Voxel-based morphometry analysis showed that expanded CAG repeat size was negatively associated with volume loss in the right cerebellar lobule IV_V and right cerebellar lobule VI using multivariable-adjusted models, controlling for potential effects of age, sex, and TIV. The results were displayed using the xjView toolbox. Color bar represents *t* values. Statistical tests were evaluated at a significance level of *P* < 0.05, the family-wise error corrected at the cluster level.


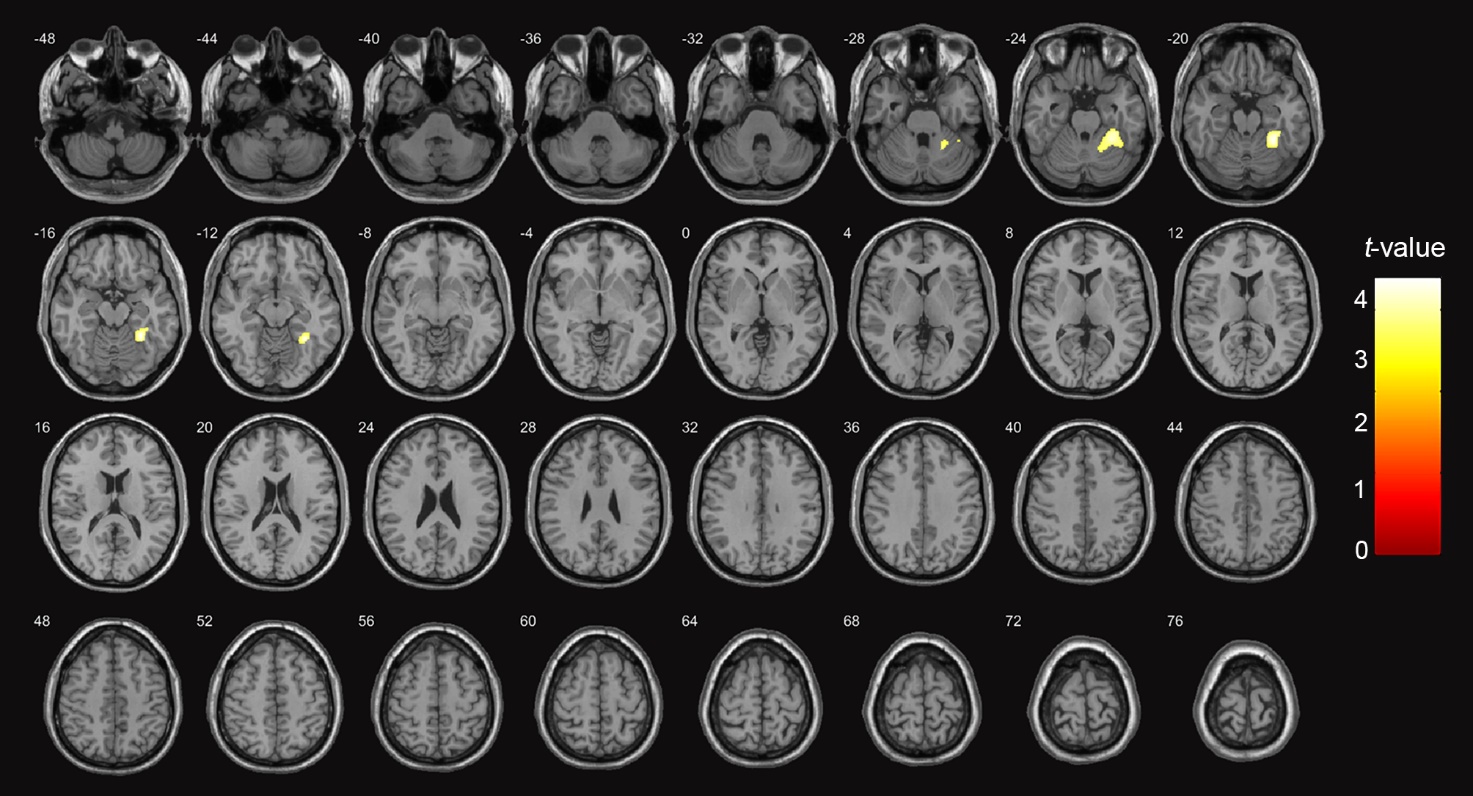

Supplement: Supplementary file 1 — Additional file 1. [file 13023_2025_3531_MOESM1_ESM.docx]
